# Exploring brain health knowledge and practices in young adults in Cuba: Dataset

**DOI:** 10.1016/j.dib.2025.111479

**Published:** 2025-03-17

**Authors:** Yunier Broche-Pérez, Diego D. Díaz-Guerra, Marena de la C. Hernández-Lugo, Zoylen Fernández-Fleites, Carlos Ramos-Galarza

**Affiliations:** a Prisma Behavioral Center, Florida, USA; b Psychology Department, Universidad Central Marta Abreu de Las Villas. Villa Clara, Cuba; c Centro de Investigación MIST, Facultad de Psicología, Universidad Indoamérica, Quito, Ecuador

**Keywords:** Brain health, Knowledge, Practices, Young adults, Cuba

## Abstract

Understanding brain health is crucial, particularly in the context of emerging public health challenges. In this scenario, this dataset aims to provide insights into Cuban youth's perceptions and awareness of brain health. The database includes information from 1, 049 Cuban participants aged between 18 and 45 years. The database is the result of a cross-sectional study conducted online, between June 30 and December 12, 2022. To explore conceptions of brain health among the Cuban population, the study utilized ``The Global Brain Health Survey''. The primary aim of this survey is to examine attitudes toward brain health and to identify factors that may encourage individuals to adopt brain health practices. By identifying knowledge gaps and misconceptions, this study highlights opportunities for improving public health initiatives tailored to this demographic. Understanding the perceptions of young people is critical, as many brain disorders develop long before clinical symptoms appear. The findings aim to inform strategies that enhance awareness and prevention efforts, ultimately contributing to better brain health outcomes among Cuban youth.

Specifications Table

**Table:** 

Subject	Public Health.
Specific subject area	*Brain health knowledge and perceptions*
Type of data	Table.
Data collection	Data collection took place between June 30 and December 12, 2021, with 1, 049 individuals voluntarily completing the survey (cross-sectional study conducted online). Participants did not receive any financial compensation..
Data source location	The data were obtained from the Cuban population, with participants from all provinces of the country. Data collection was conducted after receiving approval from the Ethics and Human Research Committee of the Department of Psychology at the Central University Marta Abreu of Las Villas.
Data accessibility	Repository name: Mendeley Data Data identification number: DOI: 10.17632/wf8dgfd6y8.3 Direct URL to data: https://data.mendeley.com/datasets/wf8dgfd6y8/3 Instructions for accessing these data: Open access database.
Related research article	*none.*

## 1 Value of the Data

• Public Health Insights: By focusing on Cuban youth's perceptions of brain health, the dataset provides valuable information that can inform public health initiatives aimed at improving mental and cognitive well-being in this demographic.
• Identification of Knowledge Gaps: The data helps identify misconceptions and areas where young people may lack information, allowing for targeted educational strategies that promote healthier behaviors.
• Informing Policy and Strategy: The findings can guide policymakers and health organizations in developing strategies that effectively address brain health concerns, particularly in the context of emerging public health challenges.
• Cultural Relevance: By analyzing data specific to the Cuban context, the study acknowledges and addresses cultural factors that may influence perceptions and practices related to brain health.
• Foundation for Further Research: The dataset can serve as a baseline for future studies exploring brain health among different populations or examining the impact of interventions aimed at improving awareness and prevention.
• Youth Engagement: Understanding the perspectives of young people is essential for fostering engagement in health initiatives, making them more relevant and appealing to this age group.

## 2 Background

Between June 2019 and August 2020, Lifebrain conducted the Global Brain Health Survey (GBHS) to gather insights into public perceptions of brain health and openness to adopting lifestyle changes for improved brain care [1]. The survey was administered online and offered in 14 languages to enhance accessibility. Its primary aim is to explore the general public's interest in brain health, including motivations for preventing brain diseases, willingness to engage in various brain health activities, and the types of support needed to facilitate lifestyle changes [2]. Respondents were also asked to identify which public health measures they believe are most effective in promoting brain health.

In this context, our dataset provides empirical evidence regarding how Cuban youth perceive and understand brain health. By examining the factors individuals associate with the development of brain diseases, this study aims to identify knowledge gaps and opportunities for enhancing public health initiatives. The findings will deepen our understanding of how young adults in Cuba view brain health risks and will inform strategies to increase awareness and prevention efforts within this demographic.

Understanding young people's perceptions of factors affecting brain health is crucial for several reasons. First, brain disorders, such as neurodegenerative diseases and cognitive impairments, often begin developing long before clinical symptoms manifest [3]. By assessing what young adults know about these factors, we can design targeted educational strategies that promote healthy behaviors and early prevention. Second, identifying misconceptions and gaps in knowledge can help correct misinformation, which is vital for improving brain health outcomes and ensuring that preventive measures are effectively communicated and adopted [2,4].

Through this dataset, we aim to gauge the level of knowledge about brain health among a representative sample of Cuba's youth. Key questions that can be addressed through this data analysis include: What factors do young people associate with the onset of brain diseases? What daily activities do they practice to protect their brains? What is their willingness to undergo testing for the early detection of brain diseases? The results can inform the design of preventive actions that promote brain health and help mitigate the prevalence of common brain-related disorders.

## 3 Data Description

The database includes information from 1, 049 Cuban participants aged between 18 and 45 years (M = 24.4, SD = 6.49) [5]. The sample predominantly consists of women (66.0 %), and most respondents have a maximum educational level of High School (57.6 %). A total of 72.6 % of participants are categorized as either students (40.0 %) or full-time workers (32.6 %) (Table 1). The survey was answered by participants from all provinces, although the distribution showed a predominance of residents from the central region of the country (Villa Clara (40.1 %), Cienfuegos (16.2 %), and Sancti Spíritus (12.4 %).

**Table 1:** Demographic characteristics of the sample (n₌1049).

Variable	Fre.	%
**Age**		
18-25	738	70.4 %
26-35	220	21.0 %
36-45	91	8.7 %
**Gender**		
Female	692	66.0 %
Male	357	34.0 %
**Education**		
Primary education	2	0.2 %
Middle School	11	1.0 %
High School	604	57.6 %
University Degree	432	41.2 %
**Employment**		
Full time employment	342	32.6 %
Full time student	420	40.0 %
Housewife	186	17.7 %
Unemployed	43	4.1 %
Volunteer work	52	5.0 %
Disability (can't work)	6	0.6 %

*Note*. Frec. (frequency)

In the dataset the self-rated health variables are also explored. In response to the question ``How would you evaluate your mental health?'' 51 % of participants self-rated their mental health as ``excellent'' or ``above average.'' Regarding self-perception of cognitive health, 68 % selected the options ``excellent'' and ``above average.'' Participants were also asked how often they thought about their brain health, to which 30.5 % responded that they did so frequently, while 28.4 % indicated that they did so rarely or never (Table 2).

**Table 2:** Self-rated health variables.

Variable	Fre.	%
**Self-rated cognitive health**		
Excellent	238	22.7 %
Above average	478	45.6 %
Average	325	31.0 %
Below average	6	0.6 %
Very bad	2	0.2 %
**Self-rated mental health**		
Excellent	167	15.9 %
Above average	372	35.5 %
Average	393	37.5 %
Below average	86	8.2 %
Very bad	31	3.0 %
**How often think about brain health**		
Frequently	320	30.5 %
Occasionally,	431	41.1 %
Rarely	238	22.7 %
Never	60	5.7 %

*Note*. Frec. (frequency)

## 4 Experimental Design, Materials and Methods

### 4.1 Study Design

The database is the result of a cross-sectional study conducted online, using Google Forms® as the platform to create the instruments and collect information from participants. Participants were recruited through Instagram, WhatsApp, and Facebook groups. Data collection took place between June 30 and December 12, 2021, with 1, 049 individuals voluntarily completing the survey. Participants did not receive any financial compensation The protocol receive approval from the Human Subjects Institutional Review Board (HSIRB) of the Universidad Central ``Marta Abreu'' de Las Villas, Department of Psychology (ethics protocol number: HSIRB-2021-016).

The sampling strategy for this online study utilized a snowball sampling method, where initial participants were recruited through social media and community networks. These participants were then encouraged to refer other eligible individuals, thereby expanding our reach within the Cuban population aged 18 to 45.

It is important to note that conducting online studies in Cuba presents unique challenges. The cost of internet access is relatively high, which can limit participation, especially among lower-income individuals. Additionally, connectivity issues are common, with intermittent access to the internet affecting the ability of potential participants to complete the study. These factors may influence the demographics of our sample and the overall response rate.

The response rate for the study was 65 %, indicating the proportion of individuals who completed the survey relative to those who were invited to participate. To encourage participation and improve completion rates, we sent 3 reminders via email and social media throughout the study.

We also monitored partial response rates, discovering that 15 % of respondents completed only part of the questionnaire. This information is critical for assessing participant engagement and will help inform strategies for future online studies.

### 4.2 Materials and Methods

To explore conceptions of brain health among the Cuban population, the study utilized ``The Global Brain Health Survey'' [1]. The survey's developers indicate that its primary aim is to examine worldwide attitudes toward brain health and to identify factors that may encourage individuals to adopt brain health practices.

The Global Brain Health Survey is an online instrument comprising 28 questions: 16 multiple-choice questions that gauge respondents’ perspectives and interests in brain health, alongside 12 demographic questions. All answers are kept anonymous, and certain questions include an “other” option to accommodate additional views or behaviors not listed. Six questions pertaining to brain health evaluations and lifestyle modifications are mandatory, although participants have the option to exit the survey at any time. The survey typically requires 15–20 min to complete.

### 4.3 Survey Measures

1. **Demographic Variables** The survey collected data on 10 demographic variables: age, gender (self-identified), education level, relationship status, healthcare experience or education, experience with longstanding illness or disability, experience as a caregiver for a family member with brain disease, participation in brain research, self-assessed cognitive health, and self-assessed mental health.
  • **Self-assessed cognitive health** was assessed with the question: *“How would you describe your ability to think, remember, and learn?”* with response options: *Excellent, Above average, Average, Below average, Very poor*.
  • **Self-assessed mental health** was assessed with the question: *“How would you describe your ability to balance your mood and emotional well-being?”* with the same response options.
  • For gender, respondents could choose from four options: *Male, Female, Other, Prefer not to answer*.
2. **Factors Influencing Brain Health**
  Respondents were asked to rate the influence of 11 factors on brain health using the question: *“In your opinion, to what extent do the following influence brain health?”* The factors included physical health, diet, and physical environment. A 5-point Likert scale was used for responses: *Very strong influence, Strong influence, Moderate influence, Weak influence, No influence*.
3. **Life Stages for Brain Health**
  Respondents were asked to indicate, *“In your opinion, at what stages in life is it important to look after one's brain?”* The life stages provided were: *In the womb (before birth), Childhood (birth to 12 years), Adolescence (13–18 years) , Young adulthood (19–45 years), Middle age (46–65 years), Old age (over 65 years)*. A 4-point Likert scale was used: *Very important, Important, Moderately important, Not important*.
4. **Brain-Related Diseases and Disorders**
  Respondents were asked to identify which of 13 listed diseases or disorders they associate with the brain, including 10 recognized brain-related conditions.
5. **Willingness to Take a Brain Health Test**
  To assess willingness to engage with a brain health test, respondents were asked: *“Imagine a simple brain health test to assess the risk of developing a brain disease. Would you wish to take such a test?”* The response options were: *Yes—definitely, Yes—probably, No—probably not, No—definitely not*.
6. **Willingness to Test for Unpreventable or Untreatable Diseases**
  Respondents were further asked: *“Would you take a test even if it provides information about a disease that cannot be prevented or treated?”* with the same set of response options: *Yes—definitely, Yes—probably, No—probably not, No—definitely not*.
7. **Reasons for Taking a Brain Health Test**
  Respondents who were willing to take a test (answered ``Yes'' to question 5) were asked: *“Why would you take a brain health test?”* They could select up to two reasons from the following options:
  (a) To get information about my cognitive and mental health,
  (b) To determine my risk of developing a brain disease,
  (c) To respond if I am at risk (e.g., change my lifestyle, seek counseling, or start treatment),
  (d) To prepare myself for the future (e.g., inform my family about the risk),
  (e) Other (please specify).
8. **Reasons for Not Taking a Brain Health Test**
  For respondents who indicated they would not take a brain health test (answered ``No'' to question 5), they were asked: *“Why would you NOT take a brain health test?”* They could select up to two reasons from the following:
  (a) I do not want to worry about something that may not happen,
  (b) I do not want to know about a disease that could not be prevented or treated,
  (c) I would be frightened by the result,
  (d) There is nothing I can do for my brain health anyway,
  (e) Other reasons (please specify).
9. **Likely Reactions to Brain Health Test Results**
  Respondents were asked to consider the scenario where a brain health test indicates a risk of developing a brain disease: *“What would be your most likely reaction?”* They were then presented with several possible reactions, including:
  (a) I would seek professional help (e.g., consult my doctor),
  (b) I would seek advice from family and friends,
  (c) I would seek information online/at the library,
  (d) I would change my lifestyle if required,
  (e) I would plan for the future,
  (f) *“Is there anything else you might do?”* (free-text response).
  Respondents rated each reaction using a 4-point Likert scale: *Definitely yes, Fairly likely, Fairly unlikely, Definitely not*.
10. **Brain Health Test Criteria**
  Respondents were asked to imagine a simple brain health test, similar to routine tests like blood pressure or cholesterol checks, to assess the risk of developing brain disease. They were asked to select the most important characteristics such a test should have, choosing up to three options from:
  (a) Affordable,
  (b) Quick to take,
  (c) Accurate,
  (d) Painless,
  (e) Subsidized by social security (via the GP),
  (f) Offered online with direct access to results,
  (g) Other (please specify).

Available in 14 languages—including English, Danish, Turkish, French, Norwegian, Catalan, German, Swedish, Hungarian, Ukrainian, Italian, Dutch, Chinese (simplified Mandarin), and Spanish—the Spanish version used for this study was specifically adapted to capture the nuances of the Spanish spoken in Cuba. To use the survey, the principal author of the study contacted the ``The Global Brain Health Survey'' project leader and requested permission to employ the instrument, which was granted by the lead researcher. The full version of the survey used in the Cuban study, can be accessed at the following link: https://data.mendeley.com/datasets/wf8dgfd6y8/1.

## Limitations

The dataset has several limitations that should be considered when interpreting the results. First, it relies on self-reported data, which may be subject to biases such as social desirability and recall bias, potentially affecting the accuracy of participants' responses. Additionally, the cross-sectional design of the study captures a snapshot in time, limiting the ability to draw causal conclusions about the relationships between variables or changes in perceptions over time. While the dataset includes participants from all provinces of Cuba, there may be sampling biases due to the recruitment methods, primarily through social media, which could lead to the underrepresentation of certain demographics or regions. Importantly, since the age range of participants is between 18 and 45 years, the results cannot be generalized to other age groups. Another limitation of this dataset is the gender distribution. This dataset exhibits a notable bias toward female respondents, which may impact the generalizability of the findings. The predominance of female participants could limit our understanding of brain health knowledge and perceptions across different gender identities. However, despite this bias, the data remain valuable as they provide significant insights into the perspectives of a substantial segment of the youth population. Additionally, the findings can serve as a foundational reference for future research, highlighting areas for further investigation into brain health perceptions among underrepresented groups. By acknowledging these limitations, we can better contextualize the results and encourage more inclusive studies in the future. Finally, the findings may not be generalizable to other populations outside of Cuba, as perceptions of brain health can vary significantly across different cultures.

## Ethics Statement

All participants provided informed consent before participation. The ethics committee of the Department of Psychology at Universidad Central ``Marta Abreu'' de Las Villas approved the study by the code HSIRB-2021-016. The research adhered to the ethical standards outlined in the 1964 Helsinki Declaration.

## CRediT authorship contribution statement

**Yunier Broche-Pérez:** Conceptualization, Data curation, Methodology, Supervision, Writing – review & editing, Project administration, Writing – original draft. **Diego D. Díaz-Guerra:** Conceptualization, Data curation, Writing – review & editing, Validation. **Marena de la C. Hernández-Lugo:** Conceptualization, Data curation, Writing – review & editing, Validation. **Zoylen Fernández-Fleites:** Conceptualization, Data curation, Validation. **Carlos Ramos-Galarza:** Conceptualization, Methodology, Supervision, Writing – review & editing.

## Data Availability

Mendeley DataGlobal Brain Health Survey_Cuba_Young Population (Original data).
